# Deriving personalised physical activity intensity thresholds by merging accelerometry with field-based walking tests: Implications for pulmonary rehabilitation

**DOI:** 10.1177/14799731221129286

**Published:** 2022-10-06

**Authors:** Ilaria Pina, Pauline Ndagire, Winceslaus Katagira, Lorna Latimer, Jakub Zatloukal, Bruce Kirenga, Sally J Singh, Mark W Orme

**Affiliations:** 1 Department of Respiratory Sciences, 574216 University of Leicester, Leicester, UK; 2 Centre for Exercise and Rehabilitation Science, NIHR Leicester Biomedical Research Centre-Respiratory, 4490 University Hospitals of Leicester NHS Trust, Leicester, UK; 3 Makerere University Lung Institute, Kampala, Uganda

**Keywords:** accelerometry, device-based physical activity, endurance shuttle walking test, exercise capacity, incremental shuttle walking tests, accelerometer cut points

## Abstract

During pulmonary rehabilitation (PR), patients receive individually tailored walking exercise training. The personalised nature of exercise prescription is a fundamental component of PR. Despite this, the measurement of physical activity (PA) has been limited to a ‘one size fits all’ approach and can be challenging to translate into clinically meaningful or real-world units, such as cadence. This discrepancy may partly explain the inconsistent evidence for the impact of PR on PA. It may also provide an opportunity to standardise PA assessment in the context of chronic respiratory disease (CRD) and PR, where field-based walking tests are routine measures. This technical note provides an example of how to develop personalised PA intensity thresholds, calibrated against an individual’s performance on the Incremental Shuttle Walking Test (ISWT; maximal) and Endurance Shuttle Walk Test (ESWT; sub-maximal). These are externally paced tests, with each level (speed) of the tests denoting a specific speed (intensity); ranging 1.8 km/h (ISWT Level 1) to 8.5 km/h (ISWT Level 12). From the ESWT, it becomes possible to evaluate adherence to each individual’s walking exercise prescription. Future research should explore this approach and its responsiveness to PR. It may be possible to extend this methodology with the inclusion of physiological parameters (e.g., heart rate, calorimetry, and oxygen consumption) to derive relative intensity markers (e.g. moderate-to-vigorous), accounting for individual differences in exercise capacity, under the same paradigm as PR exercise prescription.

The measurement and evaluation of physical activity (PA) using accelerometers is commonplace in chronic respiratory diseases (CRD) research. Numerous studies have evaluated changes in PA following pulmonary rehabilitation (PR), with mixed results.^1–4^ Exercise is a sub-domain of PA and PR has personalised exercise training, therefore PA, at its core.^5^ However, less attention has been given to the assessment of PA compared with exercise capacity. The Incremental Shuttle Walking Test (ISWT)^6^ and Endurance Shuttle Walk Test (ESWT)^7,8^ evaluate walking exercise capacity, allowing individualised walking exercise prescription and evaluation of change following PR. We propose an additional advantage to these tests; deriving personalised and real-world PA intensity thresholds.

The evaluation of accelerometry-derived free-living PA has been limited to ‘one-size-fits-all’ approaches, which do not account for individual differences in exercise capacity and can be challenging to translate into clinically meaningful units. This is demonstrated by the vast array of PA outcomes in the literature.^9,10^ In the same vein as personalised PA prescription during PR, it may be appropriate to ‘personalise’ PA intensity evaluation. By limiting the evaluation of PA to ‘one-size-fits-all’ thresholds risks making the data appear unresponsive to interventions, as a person’s potential to perform PA across intensities is inherently predicated on their physical capability. This is described previously through stratifying CRD populations by PA and exercise capacity.^11,12^ Studies examining this relationship in response to PR have shown that participants with higher baseline exercise capacity have greater PA response to PR than those with lower exercise capacity.^2,13,14^ The relationship between PA and exercise is strong, but this has not extended to merging these important constructs together.

By wearing accelerometers during field-based walking tests, it becomes possible to synchronise the assessment of PA and exercise capacity at the individual level. The ISWT and ESWT are measures of maximal and sub-maximal exercise capacity, respectively, used for the prescription of personalised walking exercise. The ISWT allows assessment across standardised levels denoting specific increasing externally paced speeds (intensity); ranging 1.8–8.53 km/h (Levels 1–12). By monitoring PA during these tests, it may be possible to generate easily interpretable personalised PA intensity thresholds with clear clinical application.

To illustrate the concept, we provide PA data of an individual living with post-tuberculosis lung disease, attending hospital-based PR in Uganda,^15^ performing ISWT and an ESWT wearing a waist-worn ActiGraph wGT3x-BT accelerometer (Figure 1).^16^ Data were obtained from an ongoing randomized controlled trial of PR for adults living with post-tuberculosis lung disease conducted at the Makerere University Lung Institute, Kampala, Uganda.^15^ The study received ethical approvals from the Mulago Hospital Research and Ethics Committee (MHREC1478), Kampala, Uganda and the Uganda National Council for Science and Technology (SS5105) and the University of Leicester (22349). For this technical note and for easy interpretation, cadence (steps/min) denotes PA intensity.^12^ Panel-A shows individual cadences for each ISWT level. The participant reached ISWT Level-6 (290 m), resulting in ESWT Level-9 (4.11 km/h) (Panel-B).^7^ Panel-C shows personalised cadence thresholds derived from ISWT and ESWT. In Panel-D, we can observe the time spent in free-living PA adjusted according to wear time and normalised for comparability, 7 days immediately pre- and post-PR, according to ISWT and ESWT thresholds. A consistent pattern of less time spent at greater PA intensities is seen. Following the completion of PR, the participant, based on pre-PR thresholds, displaced time in lower PA intensities with higher intensities (change of −9 min/day in PA pre-ISWT Level-1 and change of +34 min/day in PA above ISWT Level-1). Using the ESWT threshold, on average the participant spent 0 min/day in PA above the prescribed intensity (≥3.0 km/h) pre-PR and spent 5 min/day above their prescribed intensity post-PR.

**Figure 1.**
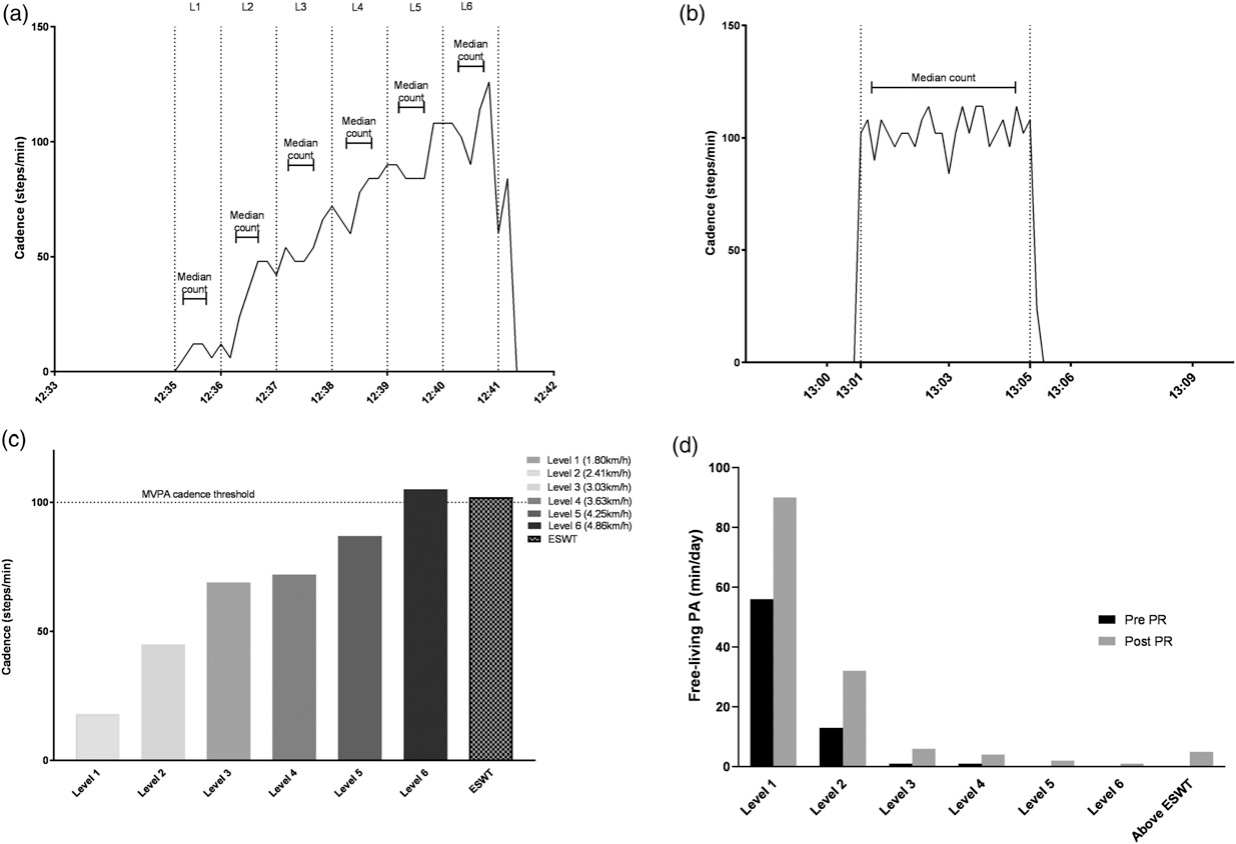
Physical activity data (cadence) of an individual living with post-tuberculosis lung disease during ISWT and ESWT. Panel A: Cadence during each level of the individual’s best ISWT. Panel B: Cadence during the ESWT (level 9; 4.11 km/h, 1.14 m/s). Panel C: Personalised physical activity intensity (cadence) thresholds derived from ESWT and each level of the ISWT. Dotted line at 100 step/min represents heuristic cadence threshold for moderate-to-vigorous physical activity.^20^ Panel D: Pre and Post PR free-living time (adjusted for wear time) spent in PA above thresholds derived from each ISWT level and above their individually prescribed speed based on the ESWT Abbreviations: PA: physical activity; PR: pulmonary rehabilitation; ISWT: incremental shuttle walking test; ESWT: endurance shuttle walk test.

We encourage future studies to explore this approach, as it adds minimal burden to the study team (e.g., no additional time for assessments needed) or participants (e.g., minimal burden wearing PA monitor during tests; similar to pulse oximeter). It is important to acknowledge that the cost of accelerometers and the additional time required for data processing remain broader practical challenges for implementation. It may be possible to extend this approach to commercial activity trackers, step-counting devices, and smart devices which people may already own or have access to.^17^ In our methodology, there appears to be a biomechanical influence due to an increase in step-width and stride variability between ISWT and ESWT thresholds, which may relate to the incremental versus steady-rate differences between the tests.^18^ Acknowledging the absence of traditional intensity thresholds (light, moderate, vigorous, very vigorous), we propose adding physiological data, such as heart rate, to derive these traditional thresholds at the individual level.

The key advantage of our proposal is its relevance to CRD populations, and other groups characterised by reduced exercise capacity, rather than using the current absolute intensity thresholds generated from healthier, younger populations with preserved exercise capacity. Whilst the ISWT and ESWT offer the most clinically relevant outputs with this new approach, it is possible to generate thresholds based on other common tests such as the six-minute walk test,^19^ but this will be more challenging to define due its self-paced nature. We have previously suggested integrating the use of PA monitors during field-based walking tests for quality assurance purposes,^16^ demonstrating further potential benefits of synchronising these methodologies. Indeed, the quality of the tests will dictate the quality (accuracy) of the resulting thresholds. Our approach, by synchronising PA assessment with the individual evaluation of exercise capacity in PR, could allow a more precise and responsive measure of changes in PA following an intervention such as PR.

In summary, we describe a new approach to deriving PA intensity thresholds, personalised to an individual’s exercise capacity using field-based walking tests, which can offer a clinically meaningful and responsive methodology to assess PA changes following PR. In anticipation of the additional advantages of synchronising PA monitoring with field-based walking tests, PA as a routine outcome measure may become more feasible in research and clinical practice.
